# Advances in Non-Thermal Processing of Meat and Monitoring Meat Protein Gels Through Vibrational Spectroscopy

**DOI:** 10.3390/foods14111929

**Published:** 2025-05-29

**Authors:** Huanhuan Li, Chenhui Li, Muhammad Shoaib, Wei Zhang, Arul Murugesan

**Affiliations:** School of Food and Biological Engineering, Jiangsu University, Zhenjiang 213013, China; justlch@163.com (C.L.); shoaib_ju@hotmail.com (M.S.); 2212318076@stmail.ujs.edu.cn (W.Z.); chemarul91@gmail.com (A.M.)

**Keywords:** meat protein, non-thermal processing, spectroscopy, chemometrics

## Abstract

Meat is a vital source of high-quality proteins, amino acids, vitamins, and minerals essential for human health. Growing demand for healthier lifestyles and technological advancements has heightened the focus on meat products, whose quality depends on meat protein properties, such as texture, water holding capacity (WHC), and structural integrity. Non-thermal processing technologies are gaining attention for enhancing the gelation properties of meat protein gels (MPGs) by optimizing solubilization, denaturation, and aggregation while preserving nutritional and sensory qualities and avoiding the drawbacks of thermal treatments. This review focuses on advanced non-thermal processing techniques, including high-pressure processing (HPP), pulsed electric fields (PEFs), ultrasound, and cold plasma, and their impact on MPGs. It also examines vibrational spectroscopy methods, such as Fourier Transform Infrared (FTIR) and Raman spectroscopy, for non-invasive analysis of MPGs. The integration of these approaches with hyperspectral imaging and machine learning is also explored as a tool to improve quality control and assessment.

## 1. Introduction

The growing global population and rising consumer expectations have intensified demand for high-quality meat products, which remain a key source of essential proteins, vitamins, and minerals [1,2]. According to the Organization for Economic Cooperation and Development, global meat consumption reached 35 kg per capita in 2024, highlighting the need to improve meat quality while ensuring safety and nutritional value.

**Figure 1:**
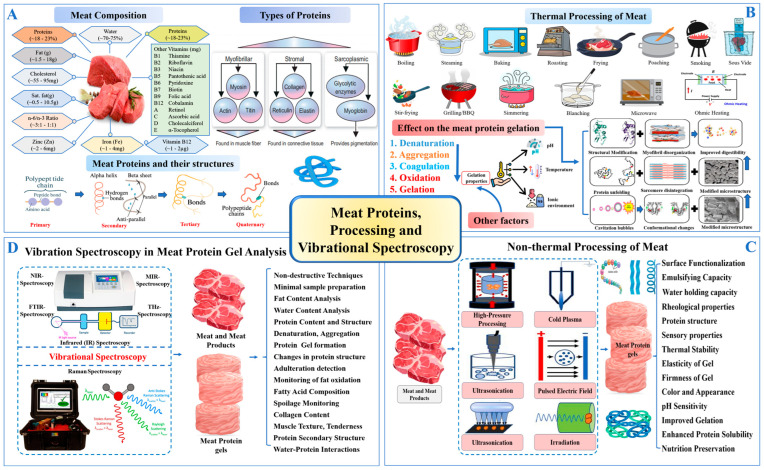
(**A**) A representation of meat composition, types of meat proteins, and their structure. (**B**) The thermal processing of meat and its effect on meat protein gel formation. (**C**) The non-thermal meat processing and its effect on meat protein gel. (**D**) The application of vibrational spectroscopy in meat protein and meat protein gel analysis.

The fundamental quality attributes of meat texture, WHC, and sensory traits are closely linked to the functional behavior of myofibrillar proteins such as myosin and actin [3] (Figure 1A). These proteins play essential roles in forming three-dimensional gel networks during thermal processing (Figure 1B). However, excessive heating can compromise their functionality [4], which has prompted increasing interest in non-thermal techniques that preserve protein structure while enhancing product quality [5,6].

Lately, various non-thermal techniques, including high-pressure processing (HPP), pulsed electric fields (PEFs), ultrasound, and cold atmospheric plasma (CAP), have been investigated for their ability to enhance the functionality of meat proteins while preserving nutritional and sensory qualities. Among these, HPP has been shown to enhance gel strength and WHC in myofibrillar proteins by promoting protein–protein interactions without causing extensive denaturation. Baldi et al. [7] and Li et al. [8] have demonstrated that PEF and ultrasound treatments can improve the texture and WHC of meat products by altering protein structures and increasing solubility. Meanwhile, CAP has emerged as a promising technique for enhancing protein gelation through oxidative modifications that increase cross-linking and protein aggregation [9]. As shown in Figure 1C, these innovative non-thermal methods help mitigate the limitations of traditional thermal treatments, offering improved texture, flavor retention, and extended shelf life while maintaining nutritional integrity. Thus, to optimize meat product quality, it is crucial to track the structural changes in proteins throughout the processing stages. Integrating advanced analytical methods with conventional processing is vital for understanding the impact on texture, WHC, and overall meat quality. Despite extensive research into individual non-thermal techniques and analytical methods, there remains a lack of comprehensive reviews addressing their combined effects on the structural and functional attributes of MPGs. In addition to vibrational spectroscopy, other viable tools for assessing protein structure include fluorescence spectroscopy, nuclear magnetic resonance (NMR), differential scanning calorimetry (DSC), and electron microscopy. Nonetheless, the synergistic use of these methods, particularly vibrational spectroscopy, for real-time protein structure monitoring has not been fully explored.

**Figure 2:**
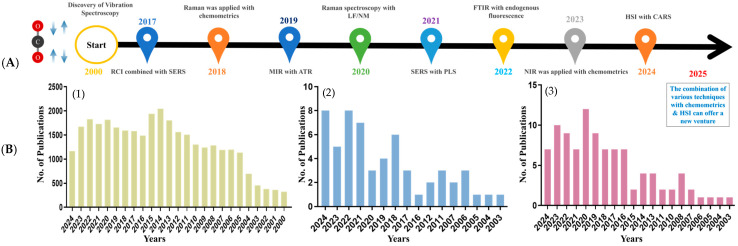
(**A**) The time-based evolution of vibrational spectroscopic techniques (2017 to 2024) [13]. (**B**) (1) Number of publications for vibration spectroscopy for meat protein analysis. (2) IR spectroscopy for meat protein analysis. (3) Raman spectroscopy for meat protein analysis. Abbreviations: RCI = Raman Chemical Imaging, SERS = Surface-enhanced Raman Spectroscopy, MIR = Mid-infrared, ATR = Attenuated Total Reflectance, LF/NMR = Low-field Nuclear Magnetic Resonance, PLS = Partial Least Square, FTIR = Fourier Transform Infrared, NIR = Near Infrared, HIS = Hyperspectral Imaging, CARS = Competitive Adaptive Reweighted Sampling.

Vibrational spectroscopy techniques, including Near-Infrared (NIR) [10], Fourier Transform Infrared (FTIR) [11], and Raman spectroscopy [12], are widely used to develop non-destructive monitoring strategies, offering a rapid alternative to conventional destructive methods such as chemical extraction or histological analysis. Significant advancements in these techniques have broadened the scope of meat protein analysis. As illustrated in Figure 2A, the timeline of advancements from 2017 to 2024 presents technological milestones, including innovations like Raman Chemical Imaging (RCI) and Surface-Enhanced Raman Spectroscopy (SERS), as well as advancements in Mid-infrared (MIR) and Attenuated Total Reflectance (ATR) methodologies. Collectively, these innovations indicate a growing trend toward applying vibrational spectroscopy in the analysis of meat proteins. Figure 2B reflects the increasing research focus for the past 2 decades, highlighting the rise in scientific publications utilizing these spectroscopic techniques, which accentuates their expanding role in enhancing meat quality assessment.

Nevertheless, the integration of vibrational spectroscopy with advanced chemometric analysis allows for precise monitoring of MPGs during processing. Advanced computational techniques, such as Partial Least Square (PLS) modeling, machine learning algorithms like Extreme Learning Machine (ELM) and Support Vector Machine (SVM), alongside deep learning models [14], are transforming the precision of predicting protein structural changes and assessing water content in meat products. These methods leverage data-driven analyses combined with spectroscopic data to significantly enhance meat quality evaluations. For instance, Nunekpeku et al. successfully employed a Long Short-Term Memory (LSTM) deep learning model integrated with fused Raman and NIR spectra to accurately predict the gel strength of minced chicken, showcasing the synergy of spectral data with advanced computational tools [12]. In a similar study, Li et al. utilized an SVM algorithm to optimize the gel quality of pork by incorporating various starches, effectively predicting gel strength and WHC, thereby demonstrating the potential of machine learning in improving the functional properties of meat gels [15].

Accordingly, this review explores recent advancements in non-thermal processing techniques, particularly focusing on methods such as HPP, ultrasound, CAP, and PEF and their impact on the structural and functional attributes of meat myofibrillar proteins. Furthermore, it highlights the use of vibrational spectroscopy techniques, such as FTIR and Raman spectroscopy, for real-time monitoring of protein gelation processes. By leveraging these non-invasive analytical methods, the industry can achieve consistent production of high-quality meat products, meeting consumer expectations for both safety and nutritional value while optimizing processing efficiency.

## 2. Role of Meat Proteins in Nutrition and Meat Processing

The functional attributes of meat proteins are key to the appeal and utility of meat and its derivatives, including their role in defining texture, flavor, and nutritional quality [16]. Meat proteins are typically categorized into three main types: myofibrillar, sarcoplasmic, and connective tissue proteins, each contributing uniquely to the quality and performance of meat products [17].

Myofibrillar proteins, which comprise about 55% to 60% of muscle proteins, are the most abundant and are critical for muscle contraction as well as MPG formation. These proteins include key structural components such as myosin, actin, tropomyosin, the troponin complex, nebulin, and titin Figure 1A. Myosin, being the most prevalent myofibrillar protein, is essential in meat gelation and WHC due to its ability to form strong, elastic gels upon thermal processing [18]. Actin collaborates with myosin to preserve structural integrity, while proteins such as tropomyosin and the troponin complex regulate muscle contraction. Additionally, nebulin and titin provide stability and elasticity to the sarcomere, forming the foundation of the muscle framework. During processing, these myofibrillar proteins undergo denaturation, exposing hydrophobic regions that promote protein aggregation. This aggregation leads to the development of a cohesive gel network, which is critical for achieving the desired texture and firmness in processed meat products.

Sarcoplasmic proteins, which are water-soluble and make up approximately 25% to 30% of muscle proteins, are critical for the biochemical and sensory attributes of meat [19]. These proteins include enzymes and pigments like myoglobin, which significantly influence meat color, a key factor in consumer acceptance. The oxidation state of myoglobin dictates the color of both fresh and processed meats. Additionally, sarcoplasmic proteins include enzymes such as proteases, which are vital in tenderization and flavor development during postmortem aging. While these proteins do not directly contribute to gel formation, they are crucial for preserving the appearance, flavor, and shelf life of meat products [20,21].

Connective tissue proteins, mainly collagen and elastin, provide structural support to muscle fibers and significantly affect meat texture and tenderness. The ratio of myofibrillar to connective tissue proteins determines gel elasticity, with higher collagen content requiring pretreatment to achieve optimal texture [22]. Collagen is abundant in connective tissues and can convert to gelatin upon heating, thereby enhancing meat tenderness and juiciness (Figure 1A). Conversely, elastin contributes to the elasticity and firmness of meat but is more resistant to thermal denaturation than collagen. The distribution and amount of collagen within meat influence its tenderness; higher collagen content is generally associated with tougher meat, especially in heavily exercised muscles. However, during cooking, the breakdown of collagen significantly contributes to the gelation process, improving the texture and WHC of certain processed meat products.

## 3. Thermal Processing of Meat and Its Impact on MP and MPG’s Structure

**Figure 3:**
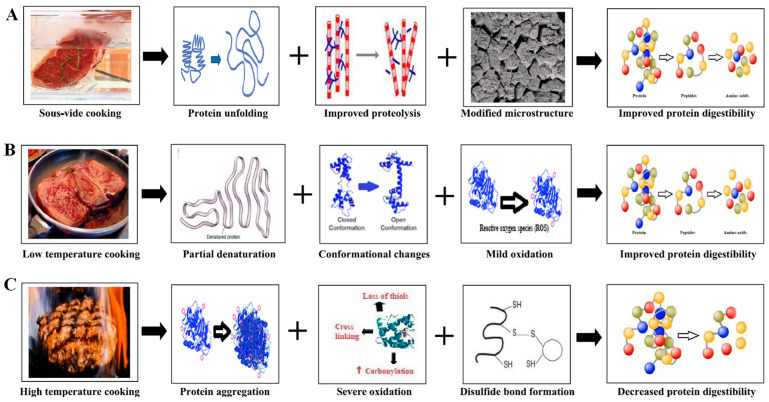
(**A**) Effect of sous-vide cooking on meat protein’s unfolding, proteolysis, microstructure, and protein digestibility. (**B**) Effect of low-temperature cooking on denaturation, oxidation, conformational and protein digestibility. (**C**) Effect of high-temperature cooking induces protein aggregation, promotes oxidation, and facilitates disulfide bond formation [28].

Thermal processing remains the cornerstone of traditional MPG formation, leveraging three key techniques: (1) heat-induced gelation, (2) chemical cross-linking, and (3) mechanical manipulation. Among these, heat-induced gelation is the most employed [23], particularly for myofibrillar proteins. The process typically involves solubilizing proteins using salts to enhance ionic strength, followed by controlled heating therefore causing the proteins to denature, exposing hydrophobic regions that promote aggregation and gel formation [24,25]. However, while effective, this method can result in over-denaturation if heating is excessive, leading to brittle gels with reduced WHC, undesirable textures, and diminished digestibility. As demonstrated in Figure 3, non-uniform heating may further compromise gel consistency, affecting the structural integrity and mouthfeel of the meat products [26,27].

Chemical cross-linking, using agents such as transglutaminase, enhances gel strength and cohesiveness by creating covalent bonds between protein molecules, thus stabilizing the gel matrix [29]. However, this approach is not without challenges. While effective in improving gel stability, the use of chemical additives raises concerns regarding food safety, potential allergenicity, and regulatory scrutiny. There are also implications for consumer perception, as these additives may alter the natural qualities of the meat, leading to hesitancy in adoption [30].

On the other hand, mechanical techniques such as chopping, mixing, and emulsifying are widely applied to improve the homogeneity of the meat matrix, enhance protein–water interactions, and promote the formation of stable gel networks during subsequent thermal processing [31,32]. These processes physically disrupt muscle fibers, exposing hydrophobic regions that enhance protein aggregation during subsequent heat treatment. Nonetheless, excessive mechanical action can degrade product quality, resulting in rubbery or overly dense gels, which negatively affect consumer satisfaction. Additionally, the energy-intensive nature of these methods can escalate production costs, especially in large-scale meat processing operations [33].

As the demand for high-quality, sustainable, and minimally processed meat products grows, the exploration of innovative approaches to MPG formation has become increasingly critical. This shift is driven not only by technological advancements but also by evolving consumer preferences for safer, more natural, and environmentally conscious food production. Addressing these demands requires the development of processing methods that are gentler, more precise, and capable of preserving the native structure of proteins while enhancing gel functionality. The next step in this exploration focuses on leveraging novel non-thermal approaches to overcome the constraints of thermal processing, offering a pathway to optimize MPG formation and improve the quality and appeal of meat products.

## 4. Non-Thermal Processing of Meat and Its Impact on MP and MPG’s Structure

Non-thermal processing methods, such as HPP, PEF, ultrasound, and CAP, offer significant advantages in enhancing MPGs without the drawbacks of heat-induced damage [34]. These techniques not only preserve the structural integrity of meat proteins but also enhance gel formation, WHC, and texture, thereby improving the overall quality of meat products. By preventing excessive protein denaturation, non-thermal methods help maintain the nutritional value of meat, offering a healthier alternative to traditional thermal treatments while ensuring superior sensory characteristics. Additionally, packaging technologies like modified atmospheric packaging (MAP), also referred to as protective gas packaging, are employed in meat processing to extend shelf life and preserve product quality by reducing oxidation and microbial growth [35]. The following sections discussed the effect of various non-thermal processing techniques on MP and MPG’s structure.

### 4.1. Effect of HPP on MP and MPG’s Structure

HPP is an innovative non-thermal technology that leverages pressures ranging from 100 to 600 MPa to enhance the quality and safety of meat products without the detrimental effects associated with traditional thermal treatments. Unlike heat-based methods, which can lead to excessive protein denaturation and nutrient loss, HPP preserves the nutritional and sensory properties of meat while effectively inactivating microbial contaminants. This technique offers a unique advantage by maintaining the fresh-like characteristics of meat products, thereby extending shelf life and improving overall product quality.

**Figure 4:**
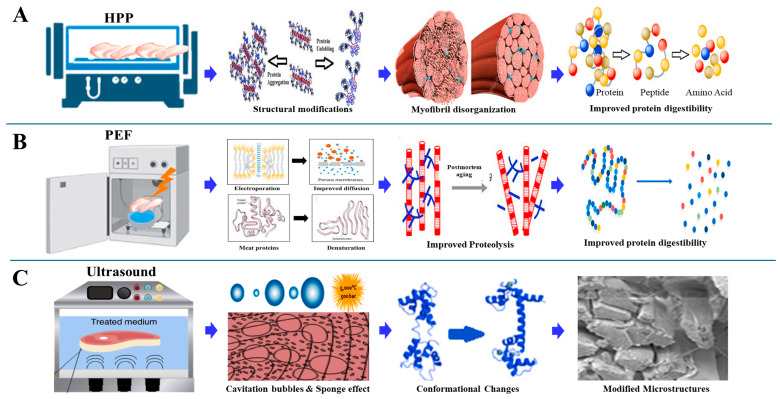
(**A**) Effect of HPP on meat protein and digestibility. (**B**) PEF improves meat proteolysis and increases meat protein and digestibility. (**C**) Ultrasound generates cavitation, induces conformational changes, and modifies microstructures, meat protein and digestibility [43].

HPP works by exerting intense pressure on meat proteins, leading to structural modifications that influence the functionality of myofibrillar proteins, which are critical for gelation [36]. At pressures above 300 MPa, there are significant alterations in the protein network, resulting in the partial unfolding and denaturation of proteins. This controlled denaturation enhances protein coagulation, aggregation, and gelation, as depicted in Figure 4A. The application of high pressure can thereby optimize meat texture, improve WHC, and enhance gel strength, which is crucial for processed products like sausages and restructured meats [37]. These structural effects of HPP on myofibrillar proteins, particularly α-helix to β-sheet transitions and enhanced gelation, are summarized in Table 1.

Moreover, HPP contributes to a notable increase in product yield of approximately 15%, as it stabilizes the gel structure of proteins and preserves the meat’s natural moisture content. This increase in yield is in stark contrast to thermal treatments, which often result in moisture loss and reduced meat yield due to excessive heat exposure [3]. HPP achieves microbial inactivation by disrupting cell membranes, altering enzyme functionality, and impairing cellular metabolic pathways, which enhances food safety without compromising quality [38]. HPP is not only effective in meat processing but also shows the potential to improve the texture and shelf life of gel-based fish products [39]. Techniques like high-pressure shift freezing and thawing have been demonstrated to prolong the storage life of frozen muscle foods while minimizing quality deterioration typically associated with conventional freezing methods [40]. Interestingly, combining HPP with natural polysaccharides has been shown to enhance the textural attributes and water-retention properties of meat gels, providing a novel approach to improving product consistency and juiciness. However, the effectiveness of HPP depends on several critical factors, including pressure intensity, processing temperature, and duration. While HPP is highly efficient at inactivating vegetative microorganisms, it requires pressures above 600 MPa combined with temperatures exceeding 100 °C to inactivate highly resistant microbial spores. This dual requirement poses challenges, as elevated temperatures can degrade heat-sensitive compounds like vitamins and flavor components. Therefore, integrating HPP with complementary preservation methods, such as mild heating or natural antimicrobials, is essential to ensure effective spore control while preserving product quality [41]. One limitation of HPP is its reduced effectiveness in low-moisture environments, making it less suitable for dry meat products where insufficient water content limits the impact of high pressure. Thus, while HPP is a versatile technique, its application is best suited for products with moderate to high water content, where it can maximize both safety and quality without compromising the nutritional integrity of the meat [42].

### 4.2. Effect of PEF on MP and MPG’s Structure

PEF technology has emerged as a non-thermal alternative that enhances the functional properties of meat proteins without the drawbacks associated with heat-based treatments [44,45]. Through the application of short, high-voltage electric fields, typically between 10 and 80 kV/cm, PEF triggers electroporation—a mechanism that disrupts cell membranes-leading to improvements in texture, tenderness, and WHC of meat products. This technique not only preserves the nutritional and sensory attributes of meat but also extends its shelf life by ensuring microbial safety while maintaining high product quality [46]. PEF operates through the generation of intense electric fields that permeabilize cell membranes, either reversibly or irreversibly. Reversible electroporation enables cells to regain functionality after exposure, while irreversible electroporation results in permanent membrane disruption, causing intracellular content leakage and microbial cell inactivation. At higher field strengths (10 to 50 kV/cm), PEF effectively inactivates microorganisms with minimal adverse effects on meat proteins compared with conventional thermal methods [47]. The ability to inactivate microbes while preserving protein structures makes PEF particularly appealing for enhancing gel formation in MPGs [48].

Notably, lower field strengths (0.5 to 5 kV/cm) are utilized to enhance mass transfer processes during freezing, drying, and osmotic dehydration, thereby improving the functional attributes of meat [7]. This enhancement is due to the microstructural changes induced by PEF, which ionizes carboxyl and amino groups in proteins, disrupting peptide interactions. This electroporation effect promotes protein unfolding, aggregation, and the reorganization of secondary and tertiary structures, thereby improving the gelation properties of myofibrillar proteins [49,50,51]. As depicted in Figure 4B, PEF causes significant modifications in protein structure, leading to changes in protein network formation and gel strength. The ionization and unfolding of protein molecules facilitate the exposure of hydrophobic regions, which enhances protein–protein interactions essential for gel formation. This not only improves the gel’s texture but also optimizes the water-binding capacity, contributing to superior product quality. Additionally, PEF has shown promise in applications beyond microbial inactivation. For instance, optimized PEF treatments have proven effective for tasks such as enzyme inactivation, dehydration, and bioactive compound extraction while maintaining the aroma, color, and nutritional value of meat products [52]. This versatile approach demonstrates that PEF can improve both the functional properties and sensory quality of meat, establishing its importance in contemporary meat processing. Table 1 further illustrates how PEF alters protein solubility and gel-forming ability through localized membrane disruption and moderate structural unfolding.

Recent developments have investigated the combined application of PEF and low-frequency magnetic fields (LF-MF) as a strategy to improve the quality of myofibrillar protein gels. LF-MF treatment, combined with pH adjustments, has been found to modify protein structures by increasing α-helix and β-sheet formations, which enhances the WHC and gel strength of pork proteins. This approach could reduce reliance on chemical additives like phosphates, which are traditionally used to improve meat quality [53].

### 4.3. Effect of Ultrasound on MP and MPG’s Structure

Ultrasound treatment has emerged as a powerful non-thermal processing technique to enhance the structural and functional properties of MPs and MPGs [54]. It operates through high-frequency sound waves that induce mechanical and biochemical changes, significantly improving protein unfolding, gel strength, WHC, and texture [55]. This non-invasive technique optimizes meat quality while preserving the nutritional and sensory characteristics, making it a promising alternative to conventional thermal treatments. Studies have demonstrated that ultrasound can enhance protein solubility, denaturation, and aggregation [56], which are critical for effective gel formation. For instance, Saleem and Ahmad investigated the impact of ultrasonication on the secondary structure and heat-induced gelation of chicken myofibrils, noting enhancements in gel strength and texture [57]. Similarly, it was found that ultrasound treatment improved the functional properties of reduced-salt chicken breast meat batter, leading to better textural outcomes and enhanced WHC [57,58,59]. These enhancements are especially advantageous for creating healthier meat products with lower sodium levels. Low-intensity, high-frequency ultrasound (<1 W/cm^2^; 100 KHz–10 MHz) has been widely adopted for its ability to maintain food composition and structure without causing thermal damage. The cavitation effect generated by ultrasound (Figure 4C) promotes nucleation and the formation of fine ice crystals during freezing, thereby reducing freezing time and minimizing damage to muscle tissues. This aligns with findings by Alarcon-Rojo et al., where ultrasound was used to reduce freezing-induced protein denaturation, preserving meat integrity [60].

**Figure 5:**
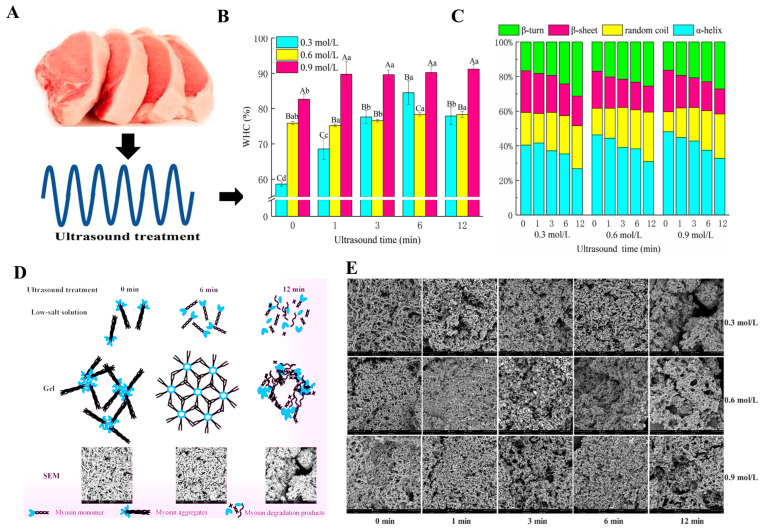
(**A**) Ultrasound treatment on pork meat. (**B**) Effects at different times on the WHC of myosin gel. Different letters (a–d) within the same NaCl concentration indicates significant difference (*p* < 0.05). Different capital letters (A–C) within the same treatment time of ultrasound indicates significant difference (*p* < 0.05). (**C**) Effects of 0.3–0.9 mol/L NaCl concentration and illustration of how ultrasound treatment on myosin gel (**D**) and potential effects on the secondary structure of myosin gel. (**E**) SEM analysis of myosin gel [63].

This causes the nucleation of crystals and encourages the development of fine ice crystals, ultimately enhancing the freezing rate. Furthermore, the collapse of cavitating bubbles produces forces strong enough to break the existing ice crystals into small pieces, which act as new nuclei and foster secondary ice nucleation. This cavitation also enhances the heat transfer rate. Consequently, ultrasound can facilitate the formation of small, uniform ice crystals, reduce freezing-induced damage to muscle tissue, and decrease protein denaturation [60]. Furthermore, ultrasound treatment has been shown to accelerate mass transfer processes, such as brining and marination. For example, Ojha et al. investigated the use of ultrasound-assisted diffusion for sodium salt replacers, significantly enhancing the physicochemical properties of pork meat [61]. Additionally, ultrasound can improve emulsification and gelling properties, thereby reducing the need for chemical additives like phosphates. Zhao et al. demonstrated that ultrasound-assisted cooking of pork meatballs improved their texture and microstructure, enhancing product quality while reducing processing times [62]. As demonstrated in Figure 5, ultrasound treatment significantly enhances MPGs by improving WHC, optimizing protein structure, and promoting robust gel network formation.

The ultrasound-induced cavitation process not only improves water diffusion rates but also facilitates the formation of a denser gel network [62]. Ultrasound treatment promotes protein unfolding, exposing hydrophilic groups that improve water retention. This effect is particularly pronounced when higher salt concentrations (e.g., 0.9 mol/L) are used, combined with treatment times between 6–12 min, resulting in structural changes from α-helices to β-sheets, which enhance the gel’s stability. However, prolonged ultrasound treatment beyond the optimal range (e.g., exceeding 12 min) can lead to protein degradation, which weakens the gel structure and diminishes its functional properties. Sun et al.’s Scanning Electron Microscopy (SEM) analyses reveal that precise ultrasound parameters result in denser and more cohesive gel networks, confirming its efficacy in enhancing MPG quality, while overexposure causes the gel network to deteriorate [63]. Additionally, recent studies have shown that these ultrasound-assisted modifications can be effectively applied across various meat products, as summarized in Table 1, further supporting its versatility in meat processing.

**Table 1:** Effect of non-thermal treatment on meat protein of gel quality.

Method	Protein Source	Parameters	Effects	References
HPP	Whitebait	100, 200, 300, 400 MPa, 10 min, 25 °C	Improved emulsification with molecular stretching increased the gel strength (4.8 fold) and myofibrillar protein gel’s digestibility (1.8 fold). α-helix changed into β-sheet with increased elastic and viscous modulus and a decrease in the sulfhydryl groups with increased hydrophobicity of protein and dense microstructure.	[64]
HPP	Pork	150 MPa	Uniform, homogenous, low viscosity, small-sized stable protein particles with emulsifying properties, increased hydrophobicity due to protein unfolding, and high dispersion stability. Fragmentation of high molecular weight proteins (myosin) occurs.	[65]
HPP	Pork	150 MPa	High-pressure homogenization-modified soy 11S globulin enhanced cooking yield, whiteness, hardness, texture, cohesiveness, shear stress, viscosity, storage, and loss modulus of myofibrillar protein. Improved water holding capacity, gel, and rheological properties of protein with the addition of soy 11S globulin.	[66]
HPP	Pork	200, 300, 400 MPa, 4 °C, 5 min	300, 400 MPa: improved drip loss with no free water with an increased fraction trapped in the myofibrillar network with no effect on water mobility and water holding capacity. The highly dynamic protein–water system with high competition between protein and water molecules, while myosin and actin are highly affected during storage.	[67]
HPP	Pork	200 MPa, 2 °C, 10 min	Soy protein (2%) increases cooking yield, gel hardness, storage modulus, sulfhydryl groups, hydrophobicity, thermal stability, water holding capacity, and immobilized water of 1% NaCl pork meat myofibrillar protein.	[37]
HPP	Beef	100, 200, 400, 600 MPa, 4 °C, 10 min	200 MPa improved cooking loss, and 400–600 MPa improved protein structure as heavy chain dissociates, reduces the density of low molecular weight bands and increases insoluble proteins due to protein aggregation. Enhanced water holding capacity, increased hardness and breaking stress of meatballs, increased elastic modulus with decreased aerobic bacteria count. No color change after cooking, improved water content.	[68]
HPP	Beef	0.1, 100, 200, 300 MPa, 21 °C 3 min	Conformational changes at 300 MPa in myosin and actin. 200, 300 MPa: reduced actomyosin content and enhance protein digestibility and degradation through the activity of cathepsin B.	[69]
HPP	Chicken	100, 150, 200, 250, 300 MPa, 10 min	200 MPa gives maximum water holding capacity, gel strength, solubility, and decreased aggregation potential of protein, increases display of Tyr and Trp residues and solubility of actin and myosin due to unfolding of the tertiary structure of the protein. 300 MPa induces disulfide cross-linking of myosin, hydrophobicity, and improved gel properties of reduced sodium myofibrillar protein.	[70]
HPP	Crab	100, 300, 500 MPa, 20 min	100 MPa partially denatures protein and forms flexible networks and stable, flexible protein structures. 500 MPa reduces myosin denaturation, suggesting protein unfolding, reduces α-helix structure, develops β-sheet portion and increases ionic, hydrogen, and hydrophobic bonds.	[3]
PEF	Chicken	0–28 kV/cm, 80–1000 Hz	Improved the solubility of myofibrillar proteins, increased rheological properties, protein aggregation, surface hydrophobicity, and sulfhydryl groups, stable tertiary structure, and increased α-helix content.	[71]
PEF	Pork	3.8 mT, 50 Hz, 3 h, 4 °C	High pH increases the WHC and electrostatic repulsion of myofibrillar proteins and decreases free water content. α-helix unfolding led to stability, tryptophan and tyrosine residues exposed from inside and contributed to hydrogen bonding, thus increasing hydrophobic interactions, uniform network, and reduced phosphate levels, which improves tenderness and texture of meat.	[53]
PEF	Beef	99 kJ/kg, 24 h, 60 °C	Increased release of ninhydrin amino nitrogen and proteolysis led to improved protein digestion due to muscle disruption. Increased penetration of digestive juices due to pores in the membrane, thus facilitating the digestion and improved protein digestibility of the meat.	[72]
PEF	Beef	0.63–0.78 kV/cm, 88.73–112 kJ/kg, 50 Hz, 60 °C, 24 h	No effect on cooking loss increases tenderness and water holding capacity, releases the rigid myofibril structure, and aggregation of sarcoplasmic proteins that rupture fat globules, ultimately resulting in juicier ribs.	[73]
PEF	Beef	0.85 kV/cm, 110.96 kJ/kg, 50 Hz, 60 °C, 24 h	Less cooking loss and hardness, with increased soluble collagen and tenderness.	[74]
PEF	Duck	2 kV/cm, 50 Hz, 12 °C	It reduces the loss of myofibrillar protein gel by 40%, with increased WHC and surface hydrophobicity, like fresh samples. Myosin actin activities were also like those of fresh duck meat, with reduced protein denaturation during thawing, maintaining gelation and emulsification.	[75]
Ultrasound	Chicken	450 W, 20 kHz	Enhanced immobilized water and β-fold content of gels, more dense, porous, and uniform gels, enhanced texture, preheating increases myosin assembly and exposes hydrophobic groups.	[76]
Ultrasound	Chicken	750 W, 20 kHz, 2 to 4 s	1% NaCl decreases particle size, suggesting enhanced protein interaction; HIU increased water holding capacity and altered the α-helical structure to the random coil and β-sheet at 1 and 2% NaCl, respectively. β-sheet increases hydrogen bond facilitated protein–protein interactions.	[77]
Ultrasound	Chicken	165 W, 30 kHz, 30 s	UF165 reduces the loss of the elastic modulus, improves gel strength and WHC due to reduced mobility, forms an intact and homogenous myofibrillar protein gel network, and maintains primary, tertiary, and secondary protein structures and gel whiteness. Fast-freezing.	[78]
Ultrasound	Chicken	200 W, 40 kHz	Ultrasound-based slightly acidic electrolyzed water (EUT) can improve the rheological properties, gel quality, and water holding capacity and effectively maintain the gel whiteness.	[79]
Ultrasound	Pork	240 W, 20 kHz, 6 min	Enhanced fat myofibrillar protein gel properties, decreased porous gel networks, and particle size of fats. The changes in hardness, springiness, and WHC of mixed gels under different MP and fat ratios as affected by HIU are shown in Figure 1. HIU decreases the hardness of the gels at a protein/fat ratio higher than 1:5, while springiness and water holding capacity increase at a ratio < 1:10, improving texture.	[80]
Ultrasound	Pork	250 W, 20 kHz, 0 to 12 min	While improving the quality of the low-salt gel while damaging the medium and high-salt gels, prolonged ultrasound treatment causes myosin degradation by increasing its solubility. Improved texture and WHC of gel.	[63]
Ultrasound	Silver carp (*Hypophthalmichthys molitrix*)	400 W, 25 kHz, 5, 10, and 15 min	Myofibrillar protein expands via the cavitation effect, which facilitates interaction between protein and water, ultimately decreases thiol, α-helix and the random coil content and increases β-sheet and solubility. Decreased gel pores with improved gel strength and WHC.	[81]
Ultrasound	*Litopenaeus vannamei*	25–29, 74–80, 123–130 W/cm^2^, 20 kHz, 60 min	Oxidation and aggregation of myofibrillar protein by increased carbonyl and free radical contents improved gelling emulsification, rheological properties, and viscoelasticity. Decreased sulfhydryl content, with increased surface hydrophobicity, fluorescence intensity, and secondary structure of protein.	[82]
Ultrasound	*Litopenaeus vannamei*	400 W, 20 kHz, 15 min	It increased the surface hydrophobicity, gel strength, WHC, emulsifying capacity, and stability of frozen shrimp’s myofibrillar protein. The cavitation effect changes the α-helix protein structure to β-sheet and β-turn. Protein unfolding increased the sulfhydryl groups.	[8]

### 4.4. Effect of CAP on MP and MPG’s Structure

Cold atmospheric plasma (CAP) has emerged as a promising non-thermal processing technique that enhances the quality and safety of meat products while preserving their structural integrity [83]. CAP operates at atmospheric pressure and low temperatures, generating reactive plasma species that effectively inactivate pathogens without significantly affecting meat proteins’ structure or functional properties. This method is particularly advantageous in meat processing, as it maintains the product’s texture, color, and nutritional value, unlike traditional thermal treatments that often lead to excessive protein denaturation and nutrient loss. By targeting cell membranes, CAP induces oxidative stress, which disrupts microbial cells and improves the shelf life of meat products, making it a viable alternative for microbial safety [84].

**Figure 6:**
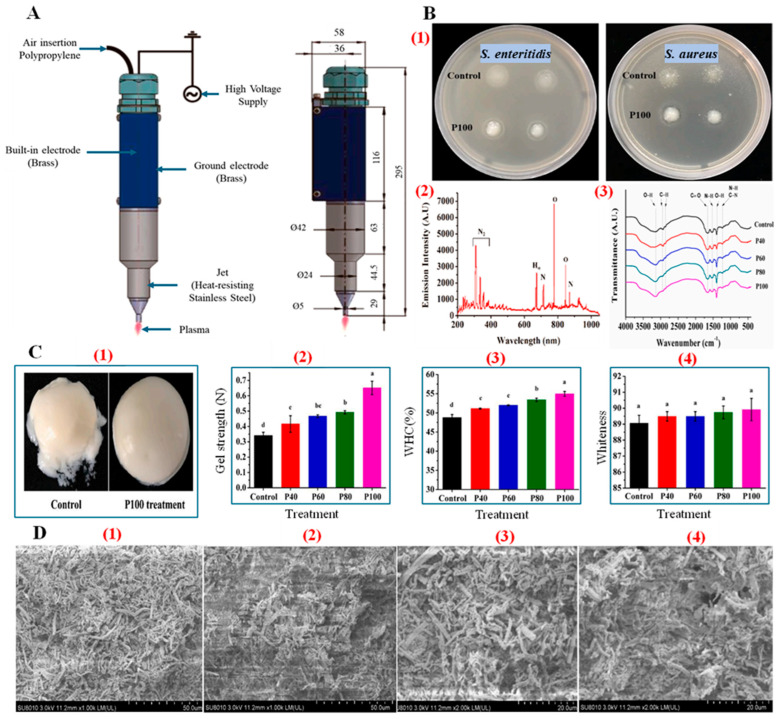
(**A**) Schematic representation of the plasma jet device [86]. (**B**) (1) Antimicrobial effects of plasma treatment on *S. Enteritidis* and *S. aureus,* showing inhibition zones for control and P100 treatment. (2) Emission spectra of the plasma, highlighting reactive species. (3) FTIR spectra of samples under different plasma treatments, showing changes in functional groups and bond vibrations. (**C**) (1) Visual comparison of gel appearance for control and P100-treated samples. (2) Gel strength comparison across different plasma treatments (P0, P40, P60, P100). (3) WHC results for each treatment. (4) Whiteness values after different plasma treatments, (a–d): different letters indicate significant differences (*p* < 0.05). (**D**) SEM micrographs of gel structure showing microstructural differences in the gel network under different plasma treatments (1, 3): Control, (2, 4): P100 treatment [85].

Recent studies have explored CAP’s application in enhancing MPGs, focusing on its ability to improve gelation properties without the drawbacks associated with heat. Qian and colleagues showed that plasma-activated water effectively enhances the gelation properties of chicken myofibrillar proteins, resulting in improved WHC and a longer shelf life. The reactive species generated by CAP, such as nitric oxide radicals, interact with meat proteins, altering their structure by increasing the formation of disulfide bonds. This results in the formation of a more compact and cohesive gel network, as shown in Figure 6, which is essential for preserving the texture and juiciness of processed meat products [85].

CAP treatment has been shown to trigger conformational modifications in myofibrillar proteins, enhancing gel strength and texture. The decrease in pH induced by plasma ions (like H_3_O^+^ and HNO_3_) leads to protein aggregation, enhancing the gel structure by promoting intramolecular and intermolecular interactions. This effect is particularly beneficial for meat products, as it strengthens the protein matrix, leading to a firmer gel that retains moisture, thereby improving the product’s overall texture and quality.

However, optimizing CAP parameters is crucial to avoid over-oxidation, which can degrade meat proteins and negatively affect gel formation [9]. Excessive exposure to reactive plasma species can lead to protein fragmentation, resulting in weaker gels with reduced water retention. Studies by Jiang et al. indicate that prolonged CAP treatment may cause protein over-oxidation, reducing gel integrity and potentially impacting the sensory qualities of meat products [87].

**Figure 7:**
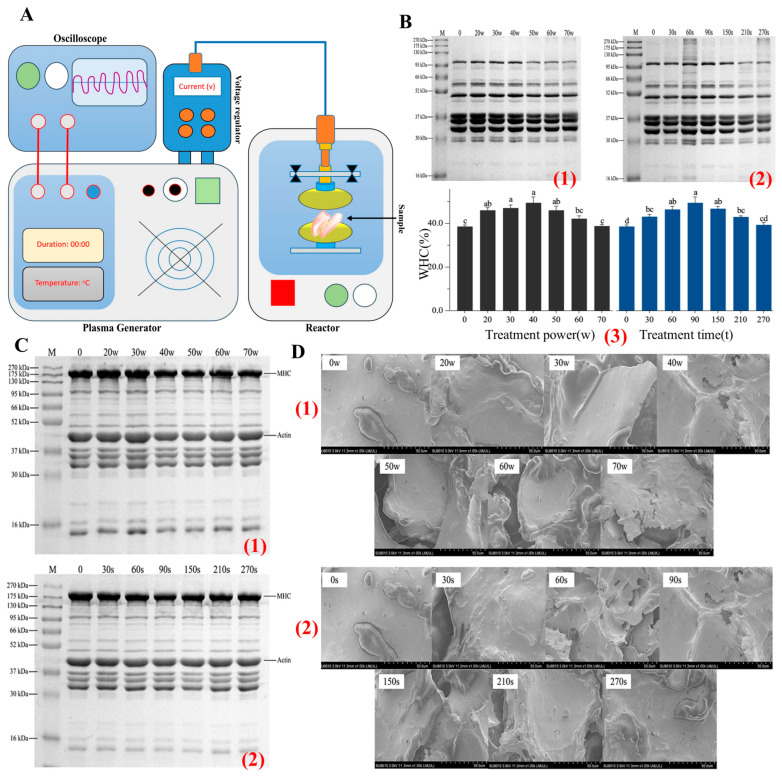
(**A**) Schematic representation of dielectric barrier discharge setup. (**B**) SDS-PAGE analysis of crude proteases from golden pomfret after CAP treatment: (1) different power levels; (2) different treatment times. (3) Water holding capacity of meat after CAP treatment, a–d: Different letters indicate significant differences (*p* < 0.05). (**C**) (1) and (2) SDS-PAGE of muscle proteins after regular pressure CAP treatment: (1) different power levels; (2) different treatment times. (**D**) SEM micrographs of plasma-treated proteins: (1) different power levels; (2) different treatment times [87].

CAP’s potential to reduce the activity of proteolytic enzymes, such as cathepsin and calpain, further supports its application in processed or aquatic meat products. For example, CAP treatment has been shown to suppress these enzymes (Figure 7), helping to stabilize protein structure and delay degradation, thereby improving texture retention and shelf life [88]. This differs from postmortem aging, where controlled protease activity is typically beneficial for tenderness development. Moreover, the use of CAP has demonstrated improvements in the gel strength of various meat products, such as sausages and restructured meats, by enhancing protein cross-linking and reducing sulfhydryl content, leading to better textural properties [89]. A comparison of CAP-induced conformational changes with other techniques is also presented in Table 1, highlighting its potential for improving gel strength while preserving nutritional quality.

However, the industrial adoption of CAP still faces challenges, including high equipment costs and the need for precise process control. Regulatory standards for food applications remain inconsistent across countries, limiting widespread approval [90,91]. Additionally, extended or suboptimal CAP treatment may affect meat aroma profiles and lead to minor sensory changes during storage.

### 4.5. Effect of MAP on MP and MPG’s Structure

**Figure 8:**
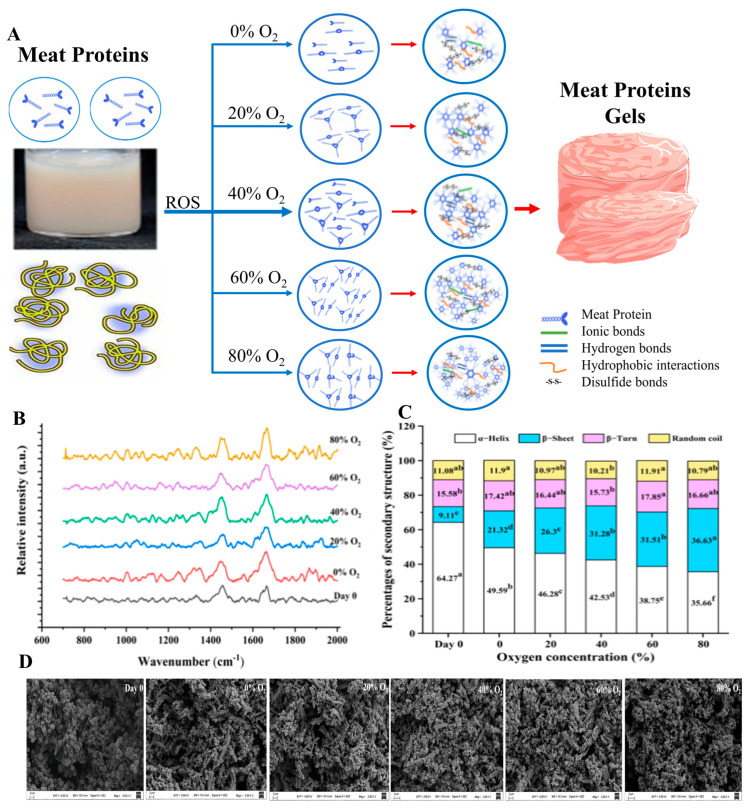
(**A**) Illustration of pork gels stored under a modified atmosphere of varying O_2_ concentrations. (**B**,**C**) Raman spectra of secondary structures of proteins, a–f: Different letters indicate significant differences (*p* < 0.05). (**D**) 1 SEM images of the microstructure of pork paste gels modified and adapted from [95].

Modified atmospheric packaging (MAP) is widely employed in the meat industry to extend the shelf life and preserve the quality of meat products by controlling the gaseous environment within the packaging [92]. This technique typically uses a combination of gases, such as carbon dioxide, nitrogen, and oxygen, to reduce spoilage and improve the visual appeal of meat [93]. High oxygen levels, often around 80%, are particularly popular for meat packaging because they enhance the redness of meat by stabilizing oxymyoglobin, thus maintaining an attractive color for consumers. Nevertheless, the benefits of high oxygen levels come with potential drawbacks, such as increased lipid oxidation, which can negatively impact the nutritional and sensory qualities of meat [94]. Recent studies have explored the effects of varying oxygen concentrations in MAP on the structural properties of meat proteins. For example, Liu et al. investigated pork treated with different oxygen levels (20%, 40%, 60%, and 80%) and found that an oxygen concentration of 60% significantly improved gelation properties by increasing carbonyl content, disulfide bond formation, and gel strength [95], as shown in Figure 8. This enhancement is attributed to the oxidative modifications that strengthen protein–protein interactions, thus increasing particle size while decreasing sulfhydryl content and protein solubility. These structural changes also involved a reduction in α-helix content with a corresponding increase in β-sheet structures, suggesting stronger hydrophobic interactions that stabilize the gel network.

Moreover, the study by Liu et al. showed that while a 60% oxygen level optimizes gelation and textural properties, a lower concentration of 40% is more effective for maintaining the redness and tenderness of pork over a 14-day refrigerated period [95]. The study also highlighted that higher oxygen levels could lead to increased carbonylation and higher shear force in sarcoplasmic and myofibrillar proteins, indicating that myoglobin-rich sarcoplasmic proteins are more prone to oxidation than myofibrillar proteins. This increased susceptibility is likely due to the localization of myoglobin in the sarcoplasm, which makes lysine residues vulnerable to oxidative changes, such as the formation of α-aminoadipic semialdehyde. Interestingly, studies have also shown that MAP can be combined with other non-thermal techniques to enhance its effectiveness [96]. For instance, Jiang et al. found that combining MAP (70% nitrogen, 30% carbon dioxide) with ultrasound treatment led to a significant decrease in β-sheet and α-helix content while improving protein solubility and digestion efficiency. This combination maintained the nutritional quality of pork meat while reducing oxidative damage. However, these findings highlight the complexity of MAP’s effects on meat proteins, as different gas compositions and processing conditions can lead to varied impacts on protein structure and functionality [97]. Despite its advantages, MAP is not without its limitations. While it extends the shelf life and maintains meat quality, the high oxygen levels can accelerate protein oxidation, leading to the formation of oxidized derivatives that may affect meat texture and nutritional value. For example, Shen et al. reported that beef steaks stored under 80% oxygen MAP exhibited increased protein carbonylation, resulting in cross-linking of lysine residues into lysinonorleucine, which may affect the meat’s tenderness and flavor [98].

There is a growing need for more research to fully understand the implications of MAP on meat protein structures, especially regarding the health effects of consuming oxidized proteins. Future studies should focus on in vivo models to explore the potential impacts of these oxidation products on human health, thus providing a more comprehensive understanding of MAP’s benefits and drawbacks in meat preservation.

### 4.6. Effect of Irradiation on MP and MPG’s Structure

**Figure 9:**
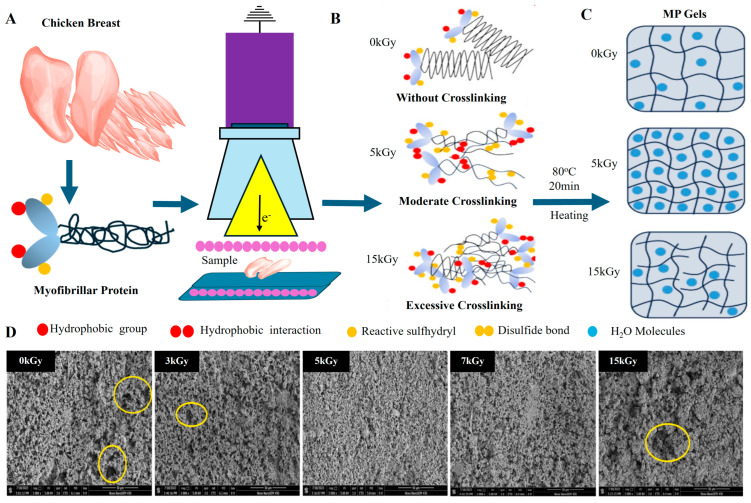
(**A**) Schematic of E-beam irradiation on chicken myofibrillar protein structure. (**B**) Protein crosslinking stages at different irradiation doses, no crosslinking (0 kGy), moderate crosslinking (5 kGy), and excessive crosslinking (15 kGy) after heating at 80 °C for 20 min. (**C**) MP gel structures represent increased network density from 0 to 15 kGy. (**D**) SEM images displaying microstructural changes in MP gels across irradiation doses [101].

Irradiation, especially through electron-beam (E-beam) technology, serves as an efficient non-thermal method to improve meat product safety and quality by prolonging shelf life and eliminating pathogens [99,100]. However, it also induces structural changes in MPs and MPGs, depending on the irradiation dose, as illustrated in Figure 9. The impact of irradiation on meat proteins is dose-dependent, with varying effects on protein crosslinking, solubility, and gelation properties. At low doses (e.g., 0 kGy), proteins maintain their native structure with minimal crosslinking and weak gelation, as they remain largely intact [101].

At moderate doses (around 5 kGy), significant improvements in protein functionality are observed. Irradiation at this level induces partial denaturation, which exposes hydrophobic and sulfhydryl groups on the protein surface. This exposure facilitates hydrophobic interactions and disulfide bond formation, enhancing protein–protein interactions and strengthening the gel network. SEM images at 5 kGy reveal a more compact and uniform gel structure, indicating better organization of the gel matrix. This enhanced network structure translates into improved WHC and gel texture as the proteins become more soluble and better dispersed in solution, which contributes to a more cohesive gel formation. Interestingly, moderate irradiation also boosts protein solubility, which is crucial for the development of strong, elastic gels with desirable textural attributes. The exposure at 5 kGy optimizes the balance between denaturation and crosslinking, resulting in improved gelation and structural integrity of MPGs. However, as the irradiation dose increases beyond optimal levels, adverse effects begin to surface. For instance, high doses (around 15 kGy) lead to excessive protein crosslinking, which results in over-aggregation and reduced solubility. SEM micrographs of meat proteins treated at 15 kGy show a more aggregated, dense, and irregular gel network with larger pores, which compromises the flexibility and WHC of the gel.

Further analysis using SDS-PAGE highlights the impact of high-dose irradiation on protein degradation [101]. At doses of 15 kGy, there is a noticeable reduction in the myosin heavy chain (MHC) band, accompanied by the formation of high-molecular-weight aggregates. These changes indicate that excessive irradiation leads to substantial protein degradation, affecting the functional properties of MPGs by reducing their ability to form cohesive gels. Therefore, optimizing irradiation doses is critical in meat processing to achieve the desired balance between microbial safety and product quality. Moderate doses (3–5 kGy) are found to enhance the structural and functional characteristics of MPGs by promoting controlled crosslinking and improving solubility, leading to better gel texture and WHC. However, excessive doses (e.g., 15 kGy) should be avoided, as they can compromise gel quality by causing undesirable aggregation and structural degradation [101]. Thus, careful optimization of irradiation parameters is essential to harness the benefits of this technology.

Despite its proven efficacy, irradiation is limited by high infrastructure costs and varying regulatory acceptance. For example, while approved in countries like the U.S. and China, it remains controversial in the EU due to labeling requirements and consumer concerns. High-dose treatments can also promote lipid oxidation, which may result in off-odors during storage.

## 5. Advances in Vibrational Spectroscopy

Non-thermal processing techniques, such as HPP, PEF, ultrasound, and cold plasma, provide a controlled approach to enhancing the structural and functional properties of MPGs while preserving the meat’s nutritional quality. However, real-time monitoring of these molecular-scale transitions conventional quality assessment methods face significant challenges [102,103].

Conventional techniques, including Texture Profile Analysis (TPA), WHC tests, and rheological measurements [104,105,106], provide macroscopic insights into the mechanical properties of MPGs, such as hardness and cohesiveness [107,108,109]. Despite their utility, these methods inherently struggle to resolve the molecular interactions that govern gelation dynamics [110,111]. This gap underscores the need for advanced, non-destructive tools capable of real-time molecular analysis, a role uniquely served by vibrational spectroscopy.

**Figure 10:**
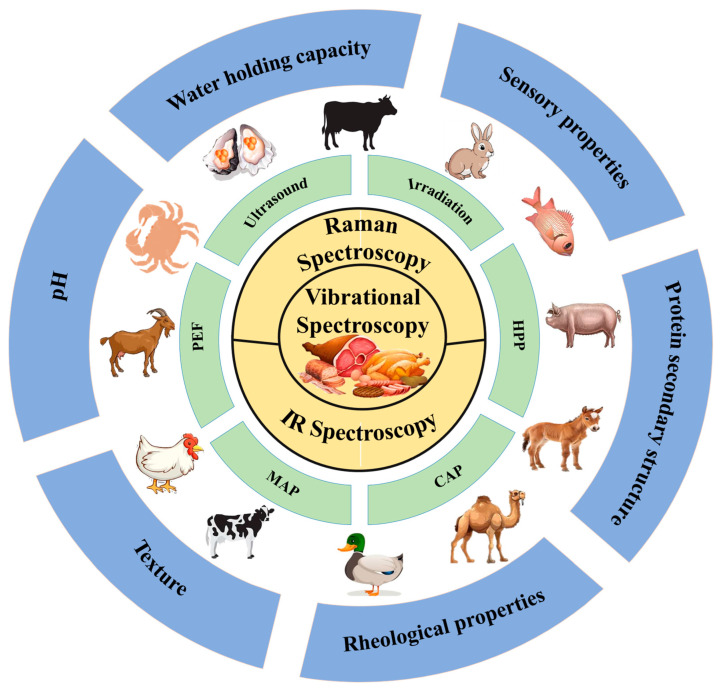
The representation of vibrational spectroscopic techniques in assessing meat properties obtained from various sources.

Vibrational spectroscopy has become a cornerstone in optimizing non-thermal food processing, primarily due to its capacity for real-time monitoring of protein dynamics—including denaturation, gelation, and aggregation—which enables data-driven process control for consistent product quality [112], as illustrated in Figure 10. As a non-destructive analytical platform, these techniques provide critical insights into molecular interactions and structural transformations, thereby driving innovation in food science to meet growing consumer demands for minimally processed, nutritionally enhanced, and safer meat products [113]. Within this technological framework, Raman spectroscopy, infrared spectroscopy, and hyperspectral imaging have emerged as particularly versatile modalities, each offering unique advantages in spatial resolution, chemical specificity, or multidimensional data acquisition during processing [114].

### 5.1. Raman Spectroscopy

**Figure 11:**
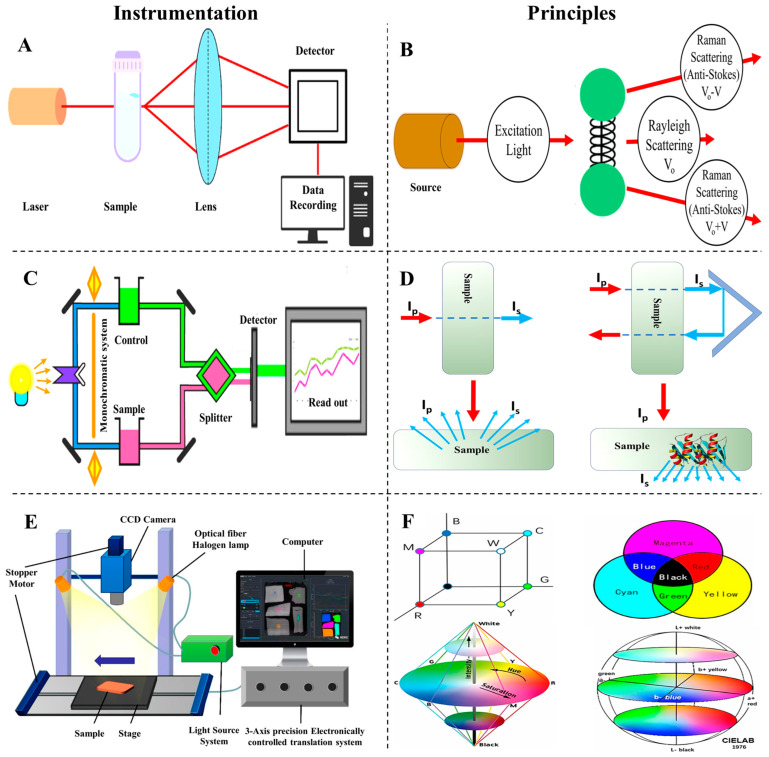
(**A**) Instrumentation and components of Raman spectrometer. (**B**) The working principle of Raman spectrometer. (**C**) Instrumentation and components of NIR spectrometer. (**D**) The working principle and mechanism of NIR. (**E**) Instrumentation and components and elements of hyperspectral imaging. (**F**) The working mechanism elements of hyperspectral imaging.

Raman spectroscopy is a vibrational technique based on inelastic light scattering, where incident photons interact with molecular vibrations, resulting in frequency shifts known as Raman shifts [115] (Figure 11A,B). These shifts provide a unique molecular fingerprint for identifying structural and chemical changes. While the majority of scattered photons are elastically scattered (Rayleigh), the small fraction that undergoes Raman scattering carries valuable structural information [116].

In the context of meat proteins, Raman spectroscopy is particularly useful for non-destructive analysis of secondary structures such as α-helices, β-sheets, and random coils. Key spectral regions, including the amide I and III bands, allow the assessment of protein folding, unfolding, and aggregation [117,118,119]. This is crucial for evaluating processing effects like heating, pH adjustment, and additive incorporation on MPGs.

For example, Raman analysis has been used to monitor the structural transition of myofibrillar proteins during gelation, revealing how changes in tryptophan and tyrosine microenvironments reflect hydrophobic interactions and crosslinking behavior [115]. In one study, incorporating 2% camellia seed oil into MP–lipid composite gels increased α-helix content and disulfide bond formation, thereby enhancing gel strength [120]. Additionally, combined Raman and NMR studies have shown that pH-driven changes in hydrogen bonding can modulate protein network formation in pork myofibrillar gels [53]. These applications highlight Raman spectroscopy’s potential as a rapid, label-free tool for monitoring molecular-level changes in meat processing and ensuring product quality.

### 5.2. Infrared Spectroscopy

Infrared (IR) spectroscopy is a widely adopted and efficient technique for rapid, non-invasive analysis in fields such as agriculture, pharmaceuticals, and food products [121,122,123]. It measures molecular vibrations based on the absorption of IR radiation by chemical bonds, producing spectral patterns that reveal the presence and structure of key functional groups. Figure 11C,D illustrate the instrumentation setup and working principle of IR spectroscopy, providing visual context for its analytical framework.

In meat protein systems, the Mid-infrared (MIR) region (4000–400 cm^−1^) is particularly informative. Key absorption bands, especially amide I, II, and III, are directly related to protein secondary structures such as α-helices and β-sheets [124]. These bands are highly sensitive to conformational changes, protein unfolding, and aggregation, making IR spectroscopy a powerful tool for monitoring myofibrillar protein behavior during processing. The use of attenuated total reflectance (ATR) further enhances its utility by allowing direct analysis of hydrated or intact samples with minimal preparation [125].

Numerous studies have demonstrated the effectiveness of IR spectroscopy in tracking protein transitions under thermal and non-thermal treatments. For example, HPP and ultrasound have been shown to induce β-sheet enrichment and reduce α-helical content, correlating with improved gel strength and WHC in MPGs [126]. These structural insights are crucial for optimizing the texture and stability of meat products. Together, these capabilities establish IR spectroscopy as a valuable technique for evaluating structural integrity and processing effects in meat protein systems.

### 5.3. Hyperspectral Imaging

Hyperspectral imaging combines spectroscopic analysis with imaging techniques, offering enhanced spatial and spectral data for assessing the quality of agricultural, food, and pharmaceutical products [127,128,129,130]. This technique captures images across hundreds of narrow, contiguous spectral bands, typically spanning the visible, NIR, and short-wave IR regions [131]. The system comprises an illumination source, an imaging spectrograph, and a detector [132]. As shown in Figure 11E, the spectrograph collects light that interacts with the sample, disperses it into its spectral components, and directs it to a detector like a CCD camera. This results in a 3D hypercube containing 2D spatial information (x and y coordinates) and 1D spectral data (wavelengths) for each pixel. The generated hypercube allows the visualization of the spectral signatures of each pixel, enabling precise identification and quantification of chemical constituents and their spatial distribution within a sample. Unlike conventional imaging systems that only capture surface characteristics, hyperspectral imaging provides a detailed assessment of internal chemical compositions, such as moisture, fat, and protein content, along with their distribution [126]. This technique is particularly beneficial for heterogeneous samples, as it overcomes the limitations of traditional spectroscopy, which only offers averaged measurements that might not fully represent the sample’s variability.

Hyperspectral imaging has demonstrated its effectiveness in various applications, including detecting defects in meat and meat products, assessing quality parameters, classifying products, inspecting poultry carcasses, and evaluating freshness [133,134]. Its ability to simultaneously analyze spatial and spectral data enables comprehensive inspection of entire surfaces, making it a powerful tool for quality control. To further illustrate the application of vibrational spectroscopic techniques in MPG analysis, Table 2 presents a comparative overview of commonly used methods, such as Raman spectroscopy, IR spectroscopy, NIR, MIR, and FTIR. The table highlights key aspects like spectral range, detection mechanisms, chemical specificity, and spectral modes, along with the benefits and constraints associated with each technique. This comparison helps clarify their roles in analyzing MPGs, especially in evaluating the effects of non-thermal processing technologies.

## 6. Application of Vibrational Spectroscopy in MPG Analysis

Vibrational spectroscopy techniques, such as Raman, IR spectroscopy, and hyperspectral imaging, provide critical advantages in characterizing MPGs by enabling non-destructive analysis of molecular interactions and structural transitions. These methods effectively decode key mechanisms, including protein denaturation kinetics, disulfide bond formation, and gel network assembly, thereby offering insights into MPG functionality such as water retention and texture modulation. By capturing real-time chemical changes during processing, vibrational spectroscopy supports the optimization of parameters for industrial applications while preserving meat’s native protein conformations. The subsequent sections first outline fundamental factors influencing MP/MPG quality, then detail how spectroscopic tools advance precision control in meat processing.

### 6.1. Characteristics and Factors Affecting MP and MPGs

The quality of meat products is heavily influenced by the structural and functional properties of MPs, and their gel-forming ability is regulated by various intrinsic and extrinsic factors [135].

One such critical factor is pH, which influences protein stability and gel-forming ability. A rapid decline in pH during post-mortem muscle conversion can disrupt the polypeptide chain network, reducing the WHC of the meat and impacting the functionality of myofibrillar and sarcoplasmic proteins. WHC influences several meat attributes, such as color, firmness, juiciness, texture, and tenderness. For instance, drip loss, which refers to the release of extracellular water as droplets on the cut surface, is a common issue linked to changes in the muscle structure post-rigor mortis, where the narrowing of filamentary space and alterations in cellular membranes [136]. Color is another vital indicator of meat quality, serving as the first visual cue for consumers. A bright red color is generally associated with freshness and consumer acceptance, particularly in red meats [137]. Factors like aging, shelf life, and moisture levels can significantly influence meat color and, consequently, its perceived quality. Meat tenderness, which is critical to consumer satisfaction, depends on the composition and structural integrity of skeletal muscle. The primary contributors to muscle strength are myofibrillar proteins and connective tissues. Collagen, the most abundant protein in connective tissue, varies in form and function across different muscle tissues. Its content and structure contribute to meat toughness. During postmortem aging, tenderness improvement is primarily due to the degradation of myofibrillar proteins, with minimal enzymatic degradation of collagen. However, interventions such as ultrasound can modify collagen structure, increasing its solubility and reducing thermal stability, thereby indirectly enhancing meat tenderness [138]. Vibrational spectroscopic techniques, such as NIR, FTIR, and Raman spectroscopy, are highly effective for assessing these properties, offering insights into meat’s chemical and physical attributes.

Overall, the gelation of myofibrillar proteins is influenced by factors such as pH, temperature, salt concentration, and mechanical processing, which regulate protein unfolding, solubility, and aggregation. These processes determine key quality attributes like water retention and texture. Advances in analytical techniques and the use of functional additives, along with clean-label and non-thermal innovations, continue to expand the functionality and applications of MP-based gels.

### 6.2. Raman Spectroscopy in MP and MPG Analysis

Raman spectroscopy is a robust, non-destructive technique widely applied in meat protein analysis. As previously discussed, Raman spectroscopy enables molecular-level analysis. In meat processing, it is particularly effective for detecting protein structural changes such as unfolding and aggregation [13]. Unlike other spectroscopic techniques, Raman spectroscopy is not hindered by water interference, making it ideal for analyzing the effects of thermal and non-thermal processing on MPGs. This capability allows for optimizing product texture, WHC, and overall quality. Additionally, Raman spectroscopy, when integrated with chemometric methods, offers rapid and high-throughput analysis, proving itself a versatile tool for assessing meat quality.

**Figure 12:**
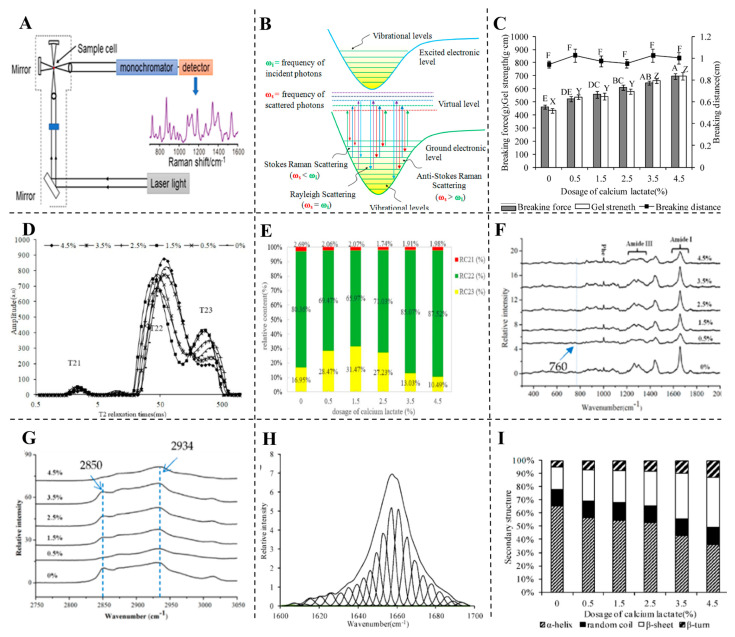
(**A**) The components of the Raman spectrometer [13]. (**B**) Representation of the Raman scattering mechanism adapted from [142]. (**C**) The effect of calcium lactate on the breaking force, distance, and gel strength. A–E: different letters at breaking force differ significantly (*p* < 0.05). X–Z: different letters at gel strength differ significantly (*p* < 0.05). F: indicates no significant difference (*p* > 0.05) at breaking distance. (**D**) The relaxation time and bound water contents. (**E**) Immobilized and free water contents. (**F**) Raman spectrum 300–2000 cm^−1^, (**G**) 2750–3050 cm^−1^, (**H**) and amide I band. (**I**) The secondary structure of proteins in surimi gels with different doses of calcium lactate adapted from [140].

For instance, combining Raman spectroscopy with low-field nuclear magnetic resonance (LF-NMR) has proven effective in evaluating the quality of surimi gels in relation to calcium lactate addition [139]. This combination provided critical information on water-binding capabilities, with results indicating that calcium lactate improves gel strength, whiteness, and WHC by influencing protein secondary structures. Calcium ions facilitate protein cross-linking, leading to a more compact gel network that significantly enhances the gelation properties of fish and meat proteins [140]. Figure 12 illustrates these structural changes, demonstrating the role of calcium in enhancing gel quality. Moreover, Raman spectroscopy has been used to study protein–lipid interactions in meat products. In a study, adding lipids led to hydrophobic interactions that enhanced gel properties, resulting in improved whiteness, WHC, and sensory attributes [141]. Further analysis showed that lipid addition modifies protein secondary structures, increasing β-sheet content, which contributes to a more stable gel network. Similarly, Wang and colleagues demonstrated that incorporating lipids into meat emulsions stabilized by chickpea protein led to changes in myosin microenvironment, enhancing water retention and oxidative stability [118].

Raman spectroscopy has also been utilized in combination with machine learning algorithms for real-time quality assessment. For example, Li’s group applied Raman spectroscopy and a deep learning model to predict pork batter quality during heating. The CNN-LSTM hybrid model excelled in predicting gel strength and whiteness, achieving high correlation coefficients (Rp > 0.93) and RPD values above 3, indicating robust predictive capabilities [143]. This integration highlights the potential of combining Raman spectroscopy with advanced algorithms to optimize meat processing quality control. Additionally, Raman spectroscopy aids in understanding the impact of non-thermal treatments on MPGs. For example, incorporating citric acid into low-salt meat products has been shown to modify protein structures, enhancing gel stability and water retention [144].

**Figure 13:**
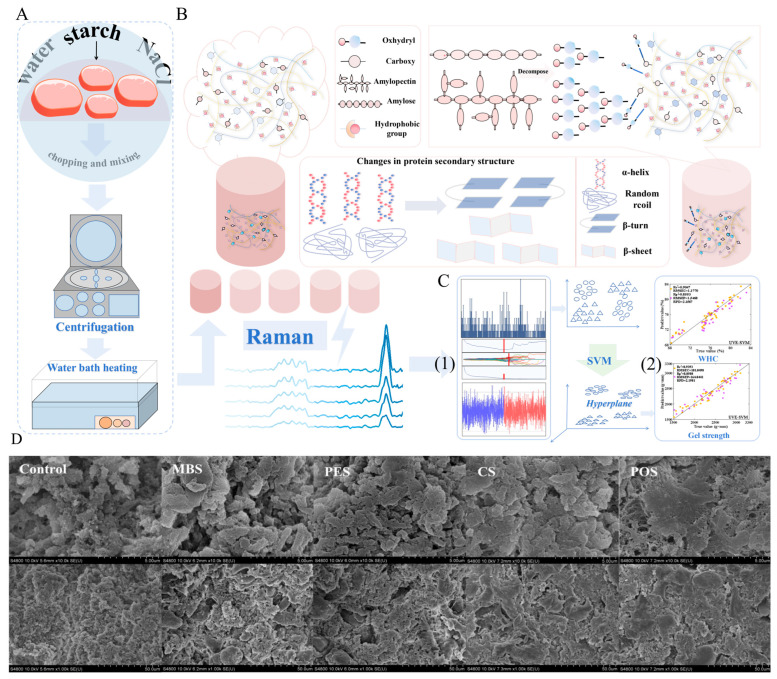
(**A**) Preparation of pork meat protein gel through mixing of starch with meat proteins, followed by centrifugation and heating. (**B**) Structural changes in proteins and starch interactions during gelation, leading to transformations in secondary structures such as α-helix, β-sheet, and random coil formations. (**C**) (1) Raman spectroscopy used to analyze the gel structure, (2) Use SVM to predict key properties like WHC and gel strength. (**D**) SEM images showing microstructural differences in gels subjected to various starch treatments (Control, MBS, PES, CS, and POS), highlighting variations in the gel matrix morphology [15].

Lastly, Raman spectroscopy’s ability to detect subtle changes in protein conformations is crucial for monitoring MPG formation. As shown in Figure 13, the gelation process involves interactions between meat proteins and additives like starch, leading to structural shifts in secondary protein elements, such as α-helices and β-sheets. Raman spectra effectively capture these alterations, highlighting the influence of processing parameters on the gel’s functional properties, such as gel strength and WHC [15].

### 6.3. Infrared Spectroscopy in MP and MPG Analysis

IR spectroscopy, encompassing techniques such as FTIR and MIR, is extensively applied in meat science to study the structural and functional attributes of proteins. By measuring the absorption of infrared light by molecular bonds, IR spectroscopy provides detailed insights into protein structures, particularly those involving amide bonds, C=O, N-H, and O-H groups. This technique is highly effective in identifying changes in meat proteins during both thermal and non-thermal processing, making it an indispensable tool for optimizing key meat quality parameters like WHC, texture, and protein gel formation. Figure 12C,D illustrate the instrumentation setup and principles behind IR spectroscopy, respectively. In meat protein analysis, FTIR is particularly useful for tracking structural changes in myofibrillar proteins. For example, studies have shown that FTIR can detect alterations in the amide I band, which correlates with shifts in protein secondary structures [145], such as increases in β-sheets and β-turns and decreases in α-helix content [146]. Additionally, FTIR with fluorescence spectroscopy has shown that dietary pterostilbene prevents the unfolding of protein structures in chicken, thereby maintaining α-helix structures. These structural changes are crucial for understanding gel formation and stability, particularly under processing conditions like high temperature, pressure or cold storage [147,148,149].

**Figure 14:**
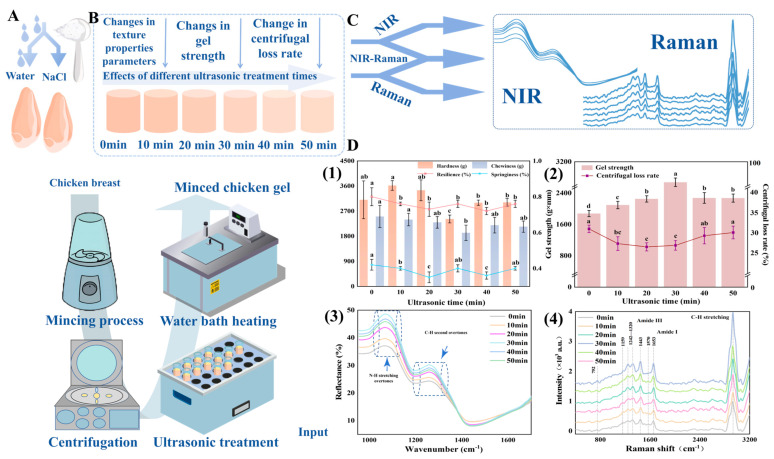
(**A**) Preparation of minced chicken gel with water and NaCl, followed by heating and ultrasonic treatment. (**B**) Effects of varying ultrasonic treatment times (0–50 min) on texture properties, gel strength, and centrifugal loss rate of minced chicken gel. (**C**) Raman, NIR, and combined NIR-Raman spectroscopy are used to analyze structural changes in the gels. (**D**) (1) Changes in hardness, chewiness, springiness, and resilience with different ultrasonic times. (2) Gel strength and centrifugal loss rate as a function of ultrasonic treatment duration. (3) NIR spectra showing changes in reflectance at different ultrasonic times. (4) Raman spectra illustrating molecular changes in the gel structure across various ultrasonic durations. Different letters indicate significant differences (*p* < 0.05) among treatments [12].

Furthermore, IR spectroscopy, combined with chemometric techniques, offers a powerful approach to analyzing the textural and structural properties of meat products [150,151]. For instance, NIR spectroscopy has been utilized to evaluate attributes like WHC, chewiness, and hardness in meat gels. The incorporation of NIR with chemometric models such as Principal Component Analysis (PCA) enhances the differentiation and prediction of textural properties [129]. Additionally, recent advancements highlight the integration of spectroscopy with machine learning algorithms for real-time quality monitoring. In practical applications, machine learning models driven by chemometric data, including Support Vector Machine (SVM) and Extreme Learning Machine (ELM), have been successfully employed alongside spectroscopic methods to accurately predict meat gel strength and texture. For example, combining NIR spectroscopy with ultrasound treatment was shown to optimize meat gel properties by modifying protein structures. The Genetic Algorithm (GA)-ELM model achieved high prediction accuracy (Rp^2^ = 0.8772), demonstrating the effectiveness of this integrated approach for non-destructive quality control in meat processing [152]. Figure 14 illustrates how ultrasonic treatment (ranging from 0 to 50 min) enhances minced chicken gel properties, including texture (hardness, cohesiveness, and springiness) and gel strength, with optimal results around 30 min due to improved protein interactions.

Moreover, combining NIR and Raman spectroscopy has demonstrated significant success in improving predictive accuracy for evaluating gel quality, as illustrated in Figure 13. Utilizing data fusion techniques and advanced models like Convolutional Neural Networks (CNNs) and Long Short-Term Memory (LSTM), these predictive approaches achieved exceptional precision [12]. This integrated approach holds significant potential for advancing the meat processing industry by enabling rapid, reliable, and non-invasive quality checks, ensuring consistent product quality while reducing reliance on labor-intensive testing methods. Chemometric techniques have shown great potential in transforming raw spectral data into actionable insights. By filtering noise, selecting critical variables, and enhancing model accuracy, chemometric-driven machine learning models support the optimization of MPGs. Table 3 summarizes current applications of chemometric analysis combined with spectroscopic techniques for meat protein analysis, highlighting advances in non-invasive quality assessment.

### 6.4. Hyperspectral Imaging in MP and MPG Analysis

Hyperspectral imaging (HSI) is an advanced, non-destructive analytical technique that integrates imaging with spectroscopy to assess meat protein properties [165,166]. By capturing detailed spectral data across a wide range of wavelengths, HSI allows for a comprehensive evaluation of multiple quality attributes, such as protein content, WHC, and structural changes, in a spatially resolved manner [167]. This capability is particularly useful for monitoring protein denaturation, texture changes, and gel formation during meat processing, providing rapid and high-throughput insights into the effects of both thermal and non-thermal treatments on meat quality [168,169]. HSI’s ability to combine spatial and spectral information makes it a powerful tool for real-time quality assessment, optimizing meat product characteristics efficiently [170].

**Figure 15:**
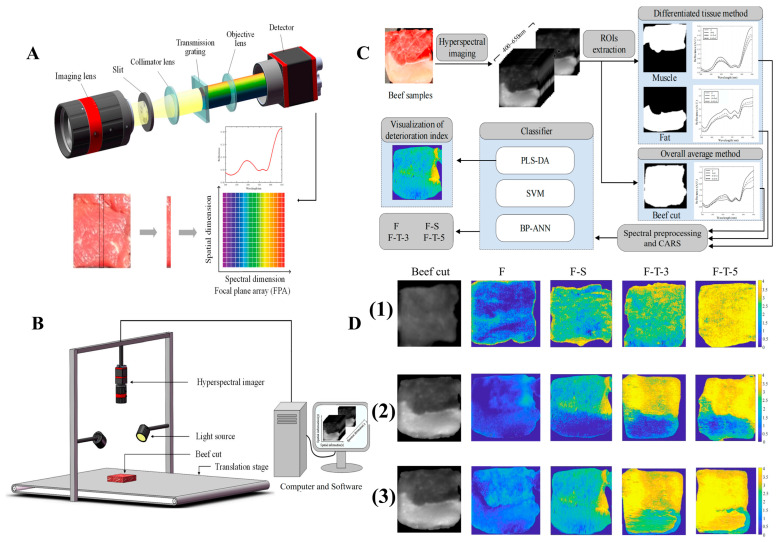
(**A**,**B**) The representation of hyperspectral imaging. (**C**) Illustrative representation of steps involved in the classification model. (**D**) Visualization of beef cut classification maps under four treatments: frozen (F), frozen-stored (F-S), and frozen-thawed for 3 days (F-T-3) and 5 days (F-T-5), based on (1) original images, (2) the overall average method, and (3) the differentiated tissue method.

Recently, HSI was employed to evaluate the freshness of frozen beef [171], combined with SVM models. The spectral images provided indices of meat deterioration by analyzing muscle tissue, offering a fast, cost-effective, and minimally invasive method compared to traditional techniques. As depicted in Figure 15, this approach efficiently assesses meat quality during storage, especially under freezing and thawing conditions. Key factors affecting quality, such as cell rupture, fat oxidation, and the denaturation of myofibrillar proteins, are closely monitored. Given that meat is primarily composed of water, proteins, fats, and carbohydrates, changes in the spectroscopic properties of these biomolecules are crucial for distinguishing between fresh, frozen-stored, and frozen-thawed samples [172,173]. In the context of meat protein analysis, HSI, alongside other spectroscopic methods like Raman and IR spectroscopy, plays a vital role. Raman spectroscopy provides detailed insights into molecular vibrations, while IR spectroscopy focuses on functional groups and protein denaturation. Together, these techniques offer a comprehensive analysis of protein structure, conformation, and interactions, which are essential for monitoring changes during meat processing. The integration of these methods can significantly enhance the ability to monitor the role of meat proteins in optimizing quality, texture, and safety across various thermal and non-thermal processing methods. This multi-modal approach ensures a deeper understanding of protein functionality, leading to improved meat processing strategies and product consistency.

## 7. Limitations and Challenges

Despite the significant advantages and diverse applications of vibrational spectroscopy in assessing meat quality, its industrial adoption faces several limitations and challenges [174,175]. One major hurdle is the complex and heterogeneous chemical composition of meat, which can lead to interference in the resultant spectrum, producing inaccurate or non-selective results. This complexity complicates sample analysis, making pre-processing essential. Combining multiple techniques, such as hyperspectral imaging and multi-spectral imaging with Raman scattering, could enhance sample detection by providing more detailed insights [176,177,178]. However, even with advancements, real-time monitoring of meat quality remains challenging. Thus, there is an urgent need for automated, on-site detection systems using spectroscopic techniques to enable real-time quality control during meat processing.

Furthermore, while non-thermal processing techniques hold great promise for maintaining meat quality, they can inadvertently cause the oxidation of biomolecules, leading to discoloration. This issue could be mitigated by optimizing the combination of non-thermal treatments to achieve the desired effects at lower intensities [179,180,181]. Additionally, the high cost of Raman spectroscopy systems remains a significant barrier to widespread adoption in the industry. Although recent developments have led to portable and handheld Raman devices, these systems are still limited in performance, making it difficult to meet the demands of large-scale production. Introducing robust, industrial-grade Raman systems on production lines would be necessary to fully realize the potential of this technology in meat processing.

Moreover, the presence of other components in meat, such as lipids, water, and carbohydrates, can interfere with the detection of subtle structural changes in proteins. Overlapping spectra from these biomolecules can complicate the interpretation of results. While chemometric techniques have been applied to address this issue, further refinement and sophistication in these analytical methods are needed to enhance accuracy and reliability [182]. These challenges highlight the need for continued research and technological advancements to fully leverage vibrational spectroscopy for industrial applications in meat quality assessment [183].

## 8. Future Prospects and Instrumentation Directions

As non-thermal technologies continue to evolve, future research must address both equipment-level innovation and process-level optimization [184]. While substantial progress has been made in improving meat protein gelation and safety through methods such as HPP, PEF, and ultrasound, many challenges remain in ensuring precise control, real-time monitoring, and industrial scalability. Instrumentation advances will be essential to bridge this gap. The increasing global demand for meat products, combined with the absence of efficient methods for in situ, rapid, precise, and high-throughput analysis, underlines the necessity for advancements in spectroscopic techniques specifically designed for the meat industry. Despite the current utility of these techniques, their limitations necessitate innovation and refinement to fully realize their potential in ensuring meat quality and safety [185,186]. Future developments in applying spectroscopy to meat processing should focus on the following areas:

1. **Enhancing real-time and accurate detection:** To achieve reliable real-time analysis, advancements in Raman spectroscopy are crucial. Incorporating chemometric methods can effectively address issues like signal overlapping and spectral misinterpretation. Employing advanced mathematical algorithms such as PCA, Partial Least Square (PLS), and SVM can optimize data extraction, leading to more precise quality assessments.
2. **Integration of visual techniques:** Combining spectroscopic methods with visual analysis offers significant potential for advancing food quality and safety assessments. Techniques like SERS [187], RCI [188], and spectral imaging [189] could be integrated to enhance tissue and microstructural assessment. This approach would not only improve monitoring of meat quality but also enhance insights into factors like animal health and nutritional status, ensuring safer meat products.
3. **Development of cost-effective, high-performance systems:** There is a pressing need to develop efficient, real-time, and in situ detection systems that are both cost-effective and high-performing. By leveraging advancements in modern optics, computer technologies, and chemometric algorithms, spectroscopy can soon provide comprehensive and accurate evaluations of meat quality, thereby elevating the standards of protein quality and safety in the meat industry.

Looking ahead, interdisciplinary collaboration across food science, analytical chemistry, and machine learning will be key to building intelligent, robust, and scalable quality monitoring platforms for the meat industry.

## 9. Conclusions

Meat proteins significantly influence the quality, texture, and nutritional value of meat products, particularly through their involvement in MPG formation. While traditional thermal processing ensures food safety, it often results in excessive protein denaturation and aggregation, adversely affecting WHC, texture, and nutritional quality. This has increased the need for alternative processing methods that preserve the functional and structural properties of meat proteins. Non-thermal techniques, including HPP, PEF, ultrasound, and CAP, have proven to be effective substitutes for conventional approaches. These technologies enhance MPG formation by maintaining the structural and functional properties of meat proteins, thereby improving texture and preserving nutritional value. To optimize MPGs further, future research should focus on fine-tuning non-thermal processing parameters, such as HPP, PEF, ultrasound, and CAP, to achieve stronger gels with enhanced WHC, cohesiveness, and stability. Crucially, such optimization requires monitoring structural changes in proteins during processing. Vibrational spectroscopy techniques, such as Raman and infrared spectroscopy (FTIR, MIR, and NIR), provide powerful tools for real-time, non-invasive monitoring of protein conformational changes, denaturation, and gelation processes. These spectroscopic methods offer critical insights that are essential for ensuring the production of high-quality meat products. Additionally, integrating vibrational spectroscopy with advanced analytical approaches such as chemometrics, hyperspectral imaging, and machine learning can significantly improve the accuracy and specificity of meat quality assessments. This integration allows for rapid, high-throughput evaluations of complex meat systems, facilitating better predictions of processing outcomes. The combined use of non-thermal processing techniques with advanced vibrational spectroscopy and complementary technologies presents a promising strategy for producing high-quality, functional meat products that meet consumer demand for healthier, minimally processed foods. Continued research and technological innovations in this area will be pivotal in enhancing the efficiency, accuracy, and sustainability of meat production and quality control systems, ultimately aligning with industry trends toward more sustainable and consumer-friendly food processing solutions.

## Figures and Tables

**Table 2:** Comparison of IR and Raman spectroscopy adapted from [126].

Technique	Spectral Region (nm)	Mechanism	Chemical Selection	Spectral Mode	Pros	Cons
Raman	2500	Inelastic scattering	Change in polarization	Reflection	Structural information and qualitative analysis, low water sensitivity	Biomolecular interference, low sensitivity, and scattering
IR	2500–25,000	Absorption	Change in dipole moment	Transmission, attenuated total reflectance	Structural information and qualitative analysis	Water and CO_2_ signal interference, not suitable for moist samples
NIR	1000–2500	Absorption	Change in dipole moment	Transmission	Low sample preparation, increased sensitivity to water, and physical structure	A reference and dry samples are needed, with low specificity and spectral overlapping
MIR	2500–25,000	Absorption	Change in dipole moment	Transmission	Clear peaks, high sensitivity	Water interference requires a dry sample with less penetration of light
FTIR	MIR-NIR	Absorption/emission	-	Transmission, reflectance	Protein secondary structure, distinct peaks	Water interference, overlapping signals

**Table 3:** Application of different spectroscopic techniques and chemometric algorithms on meat from multiple sources.

Meat Source	Technique	Chemometric Analysis	Study Findings	References
Beef	Raman	CARS-PLS	Non-destructive analysis of color, texture, and water content improved WHC.	[153]
Fish	MIR	PCA	Change in secondary protein structure through reduced band intensity of myosin after freezing.	[154]
Fish	NIR	PCA-CARS	Textural properties such as WHC, hardness, resilience, springiness, chewiness, and shear force were identified.	[129]
Beef	Raman	PLSR	Raman spectra can predict the lactic acid bacteria, pH, lightness, and viable counts in packaged beef samples packed under modified atmosphere packaging. Lactic acid was detected by PO_3_^2−^ stretching and lactate in meat.	[155]
Beef	Raman, FTIR	PLSR	Combining both techniques can detect the wavelengths for C-N stretching, tyrosine double bands, and S-S bending vibration, representing the meat spoilage with greater accuracy by suggesting the transition of β-fold to α-helix.	[156]
Beef	NIR	PLS-SVR	Total volatile nitrogen content was identified with greater prediction accuracy and stability. Peaks were identified for C–H bonds, C–O bonds, and N–H bonds in proteins, as well as for fats and water.	[157]
Beef	Hyperspectral imaging	PLS-DA	Discriminate among various meat textures by correlating collagen protein and tenderness properties.	[158]
Foal	MIR	PLS	Accurately predict moisture, collagen, lipids, color, and sensory parameters. After applying regression treatments, the discriminant analysis yielded positive results, with a spectrum range of 2198–1118 cm^−1^, corresponding to various proteins.	[159]
Bovine	Raman	PCA	Predict the hardness and stiffness in meat with accuracy; spectral peak change in the amide III band indicates changes in α-helix and β-sheet content ratios, which suggest variation in the residual composition and the protein secondary structure. The peak at 1003 cm^−1^ represents the phenylalanine’s symmetric vibration and its content.	[160]
Pork	NIR hyperspectral imaging	PLSR-SVM	The identification of protein, fat (1210, 1233, and 1238 nm), and water (972 nm) peaks in the NIR fingerprint accurately predicted intramuscular fat from hyperspectral imaging in a polythene bag.	[161]
Beef	Raman	PLS	Predict hydrophobicity of amino acid residues and secondary structural composition of proteins (α-helix and β-sheet) for identification of texture from frozen/thawed meat with variables located in 60–1060 cm^−1^, 1370–1490 cm^−1^, and 1550–1680 cm^−1^. Modeling of spectra predicted tenderness, chewiness, and hardness with greater accuracy.	[162]
Lamb, camel, beef	NIR	PCA, PLS-DA	In NIR absorbance, two isosbestic points associated with protein and moisture were seen at 1028 nm (O-H, N-H) and 1224 nm (C-H) in all samples, while PC2 at 1242 nm (C-H) and 1372 nm (C-H) indicates both lipids (fatty acid profile) and protein content.	[163]
Fish	FTIR	PCA	Protein secondary structures (sarcoplasmic, myofibrillar, and alkali-aided proteins) were identified. PCA suggested that alkali-aided proteins have β-sheet and reduced sulfhydryl content and hydrophobicity, sarcoplasmic proteins have α-helix whereas myofibrillar proteins have lipids and β-sheet	[164]

Competitive Adaptive Reweighted Sampling-Partial Least Square (CARS-PLS); Partial Least Square Regression (PLSR); Support Vector Machine (SVM); Principal Component Analysis (PCA); Support Vector Regression (SVR).

## Data Availability

No new data were created or analyzed in this study. Data sharing is not applicable to this article.
